# Supplementary material for: Four-octyl itaconate improves osteoarthritis by enhancing autophagy in chondrocytes via PI3K/AKT/mTOR signalling pathway inhibition
Source: Commun Biol. 2022 Jun 29;5:641. doi: 10.1038/s42003-022-03592-6 (PMC9242998; doi:10.1038/s42003-022-03592-6)
Supplement: Supplementary file 2 — Supplementary information [file 42003_2022_3592_MOESM2_ESM.pdf]

Four-octyl itaconate improves osteoarthritis by enhancing autophagy in chondrocytes via PI3K/AKT/mTOR signalling pathway inhibition

Xuekang Pan[1,2*], Huajian Shan[1*], Jinyu Bai[1*], Tian Gao[2], Bao Chen[2], Zhonghai Shen[2], Haibin Zhou[1], Huigen Lu[2#], Lei Sheng[1#], Xiaozhong Zhou[1#]

[1] Department of Orthopaedics, The Second Affiliated Hospital of Soochow University, Suzhou 215004, China;

[2] Department of Orthopaedics, The Second Affiliated Hospital of Jiaxing University, Jiaxing 314000, China.

[#] Corresponding to: Xiaozhong Zhou: zhouxz@suda.edu.cn; Huigen Lu: 13758076161@163.com; Lei Sheng: shenglei510@suda.edu.cn.

[*] Equal contributions: Xuekang Pan, Huajian Shan, Jinyu Bai.

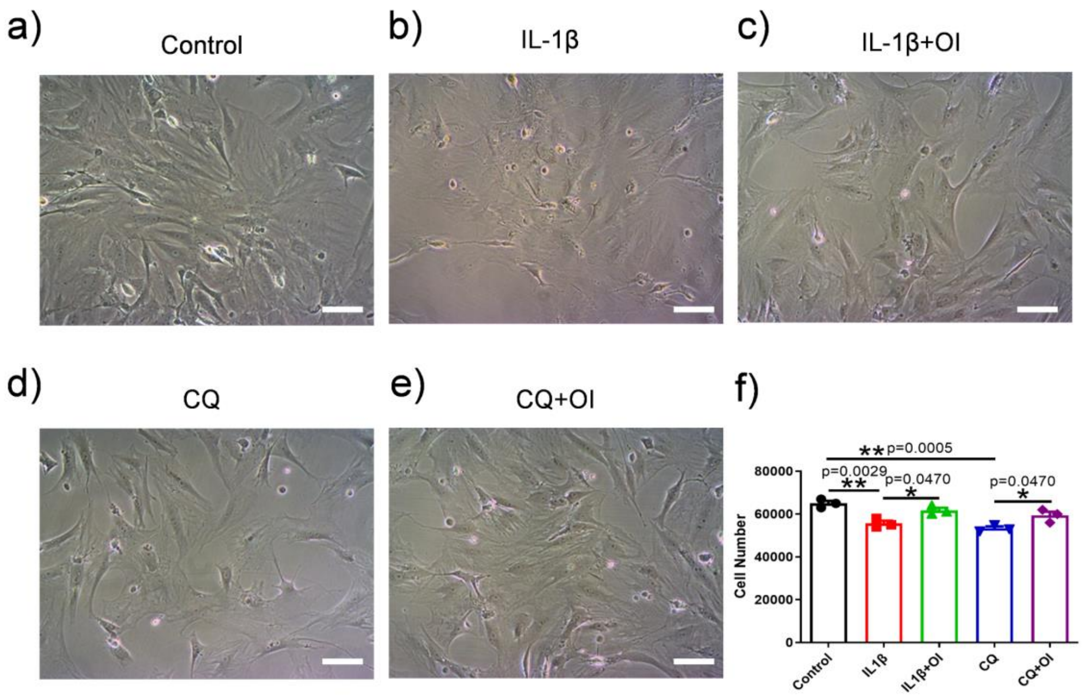

Supplementary Fig. 1 The morphological analysis of the chondrocytes in different groups (magnification, x200, scale bars: 50 μm). a) The controls, the rat primary chondrocytes with no drugs added were cultured for 48 h. **b-e)** Images of rat primary chondrocytes cultured with **b)** IL-1β, **c)** IL-1β+OI, **d)** CQ, **e)** CQ+OI for 48 h. **f)** Chondrocyte count among groups. The error bar is SD. The data are expressed as a mean ± SD (n=3), *, *P*<0.05.

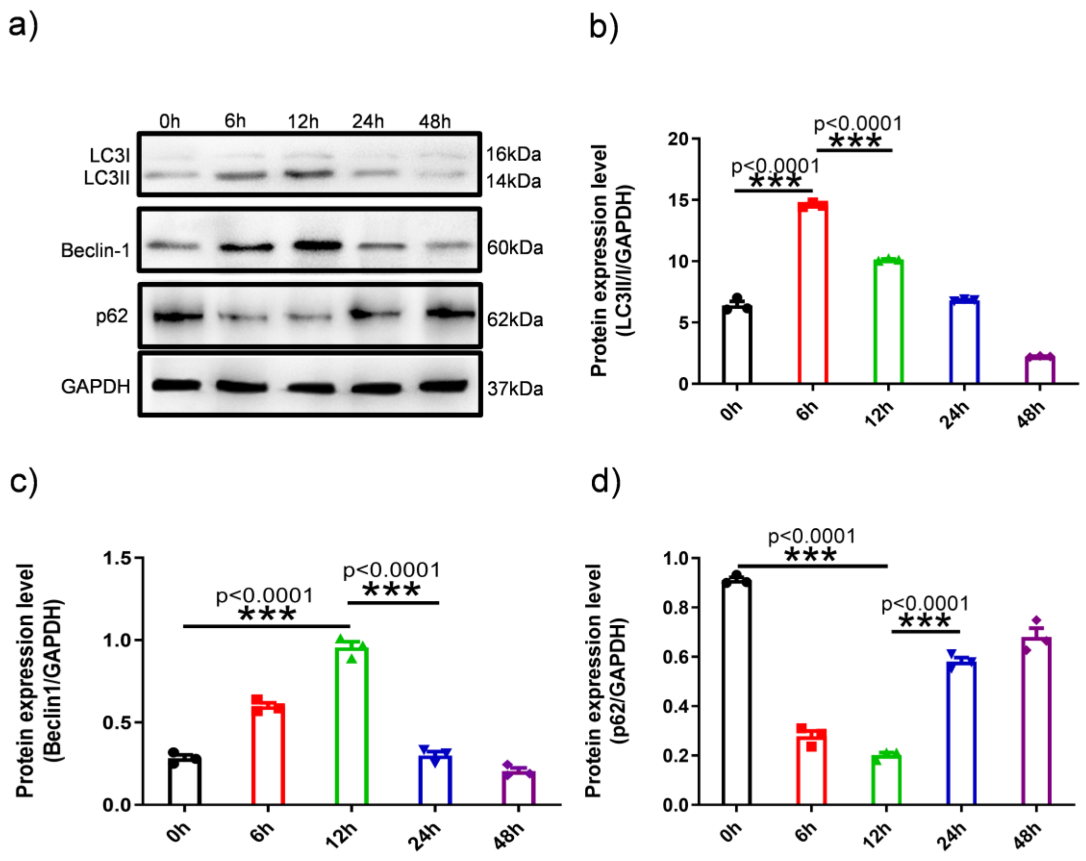

Supplementary Fig. 2 IL-1β exposure induced defection of autophagy in chondrocytes. a) A western blot analysis was carried out on IL-1β-induced C28/I2 cells at different time points (0, 6, 12, 24, and 48 h) with antibodies directed at LC3, Beclin1 and p62 and **b-d)** normalized to GAPDH. The error bar is SD. Data represent the mean ± SD (n=3), **, $P< 0.01$ and ***, $P< 0.001$.

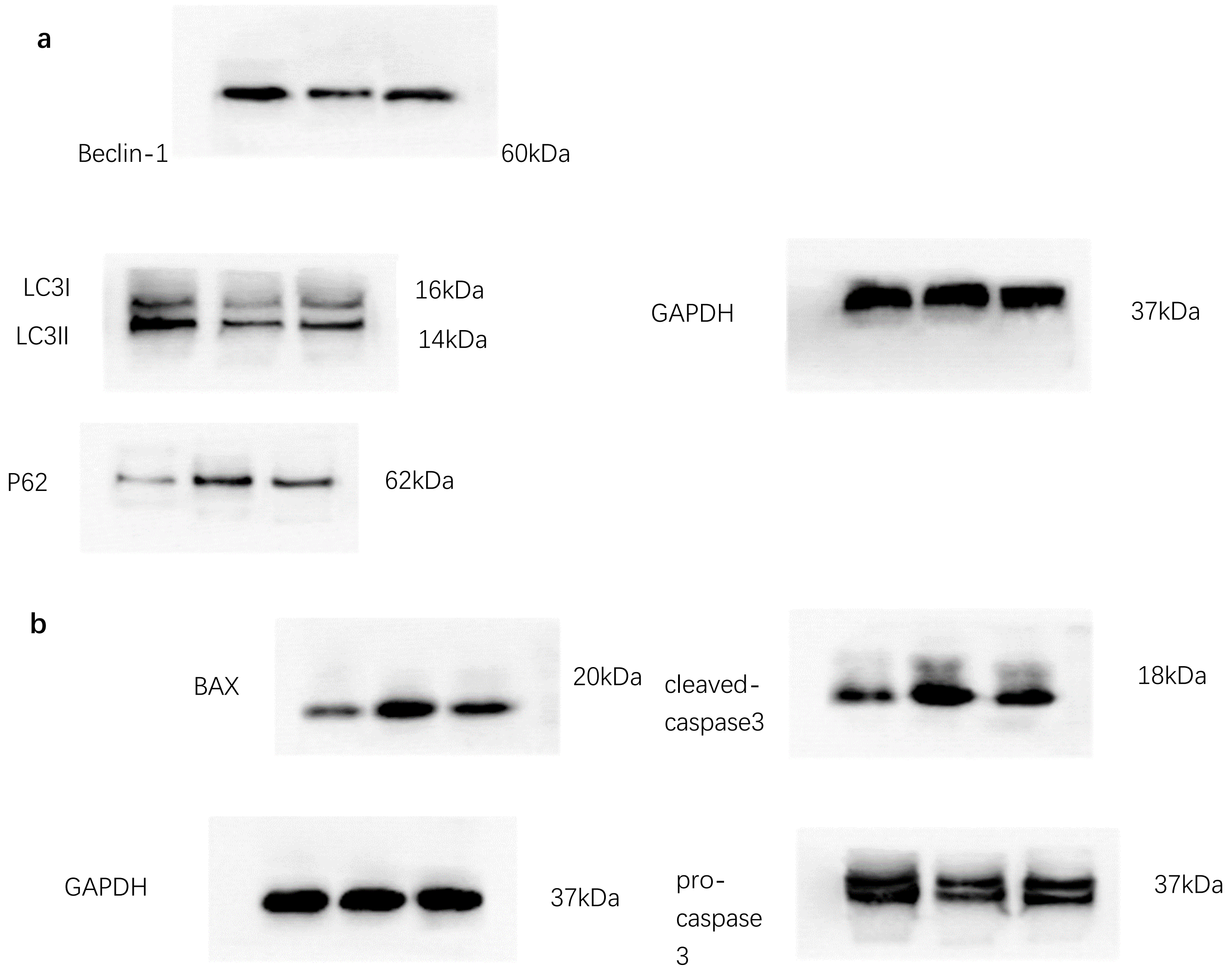

Supplementary Figure 3 – Related to Figure 2d, 2h

a Western blotting analysis of autophagy marker expression further showed that IL-1β decreased the LC3II/LC3I and Beclin-1 and increased the p62, but this effect was inhibited by OI. **b** Western blotting analysis of BAX, pro-caspase3 and cleaved caspase3 protein expression. OI inhibited cleaved caspase-3 and BAX expression, while IL-1β promoted their expression. Primary antibodies are indicated on top. GAPDH used as loading controls.

a

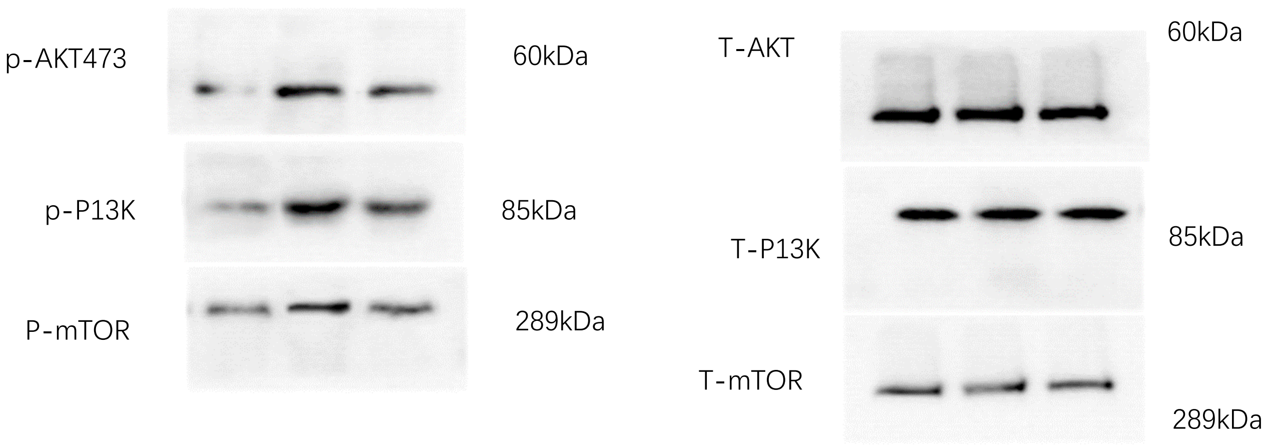

Supplementary Figure 4 – Related to Figure 3a

a Representative images of Western blotting analysis of p-AKT, T-AKT, p-PI3K, T-PI3K, p-mTOR and T-mTOR in C28/I2 cells treated with IL-1β and OI (n=3). In comparison to controls, PI3K, p-AKT, and p-mTOR levels were increased in C28/I2 cells treated with IL-1β but decreased in C28/I2 cells treated with OI. Primary antibodies are indicated on top.

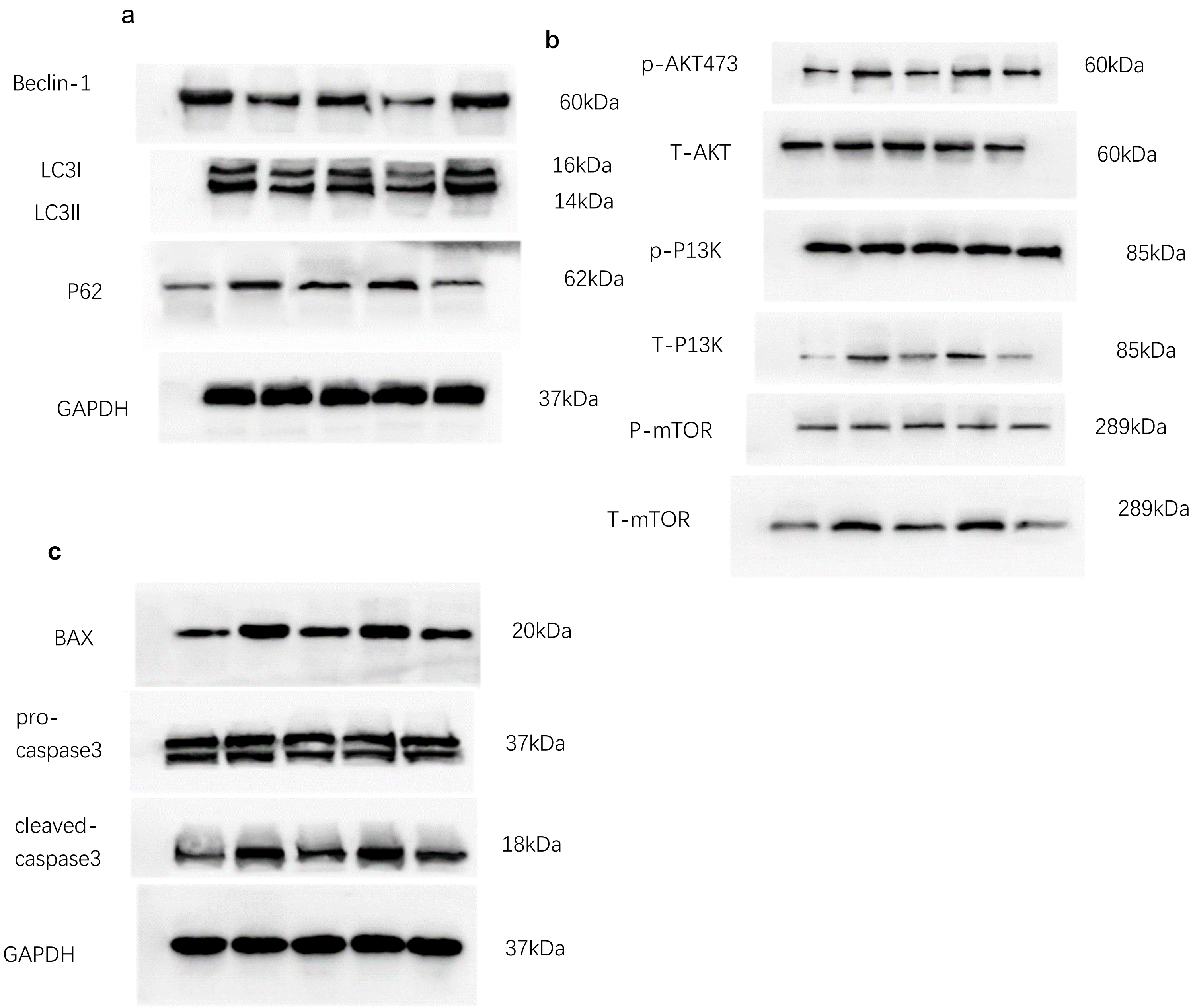

Supplementary Figure 5 – Related to Figure 4e, 4i, 4m.

a Western blotting analysis showed that compared with CQ and IL-1β, OI significantly enhanced the fluorescence intensity of Beclin1. **b** Western blot analyses showed that IL-1β and CQ markedly increased levels of phosphorylation of PI3K, AKT, and mTOR, whereas OI treatment inhibited IL-1β- and CQ-associated phosphorylation of the PI3K/AKT/mTOR signalling pathway. **c** The WB showed that OI weakened the promoting effect of IL-1β and CQ on the protein expression of cleaved caspase 3 and Bax. Primary antibodies are indicated on top. GAPDH used as loading controls.

a

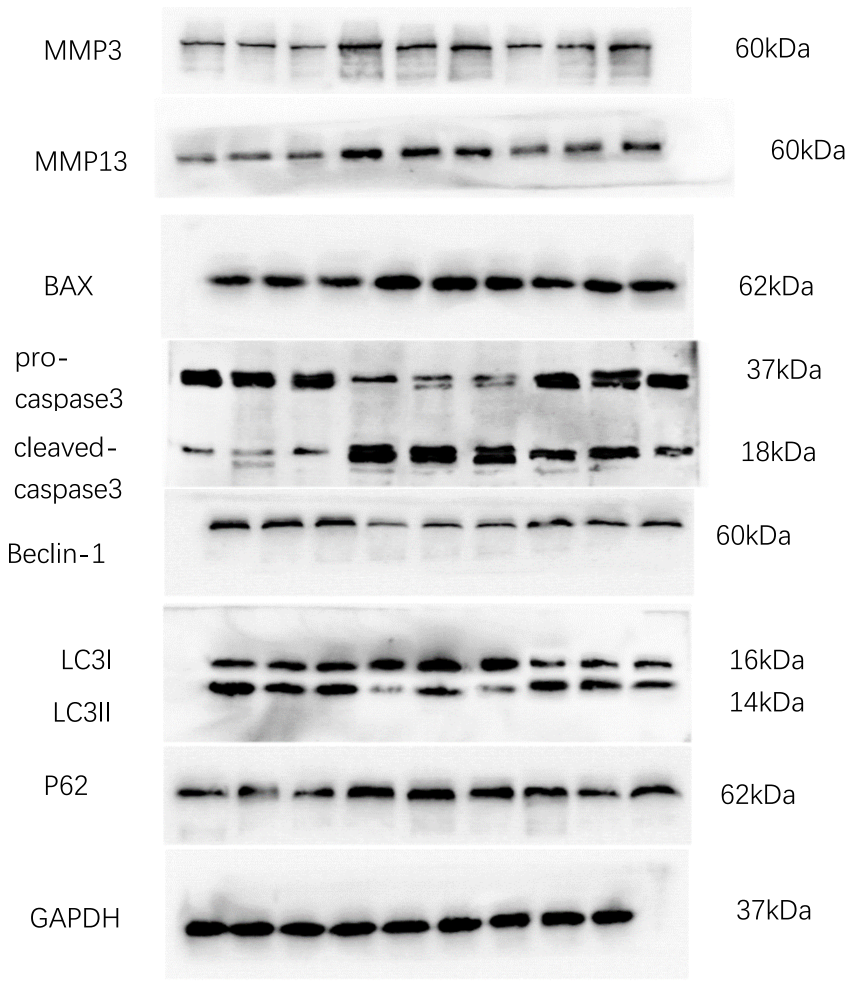

Supplementary Figure 6 – Related to Figure 7a.

a As a result of Western blotting analysis, cartilage degradation-related proteins (MMP3, MMP13) and autophagy-related proteins (LC3, Beclin1, p62) were also significantly altered. Primary antibodies are indicated on top. GAPDH used as loading controls.

a

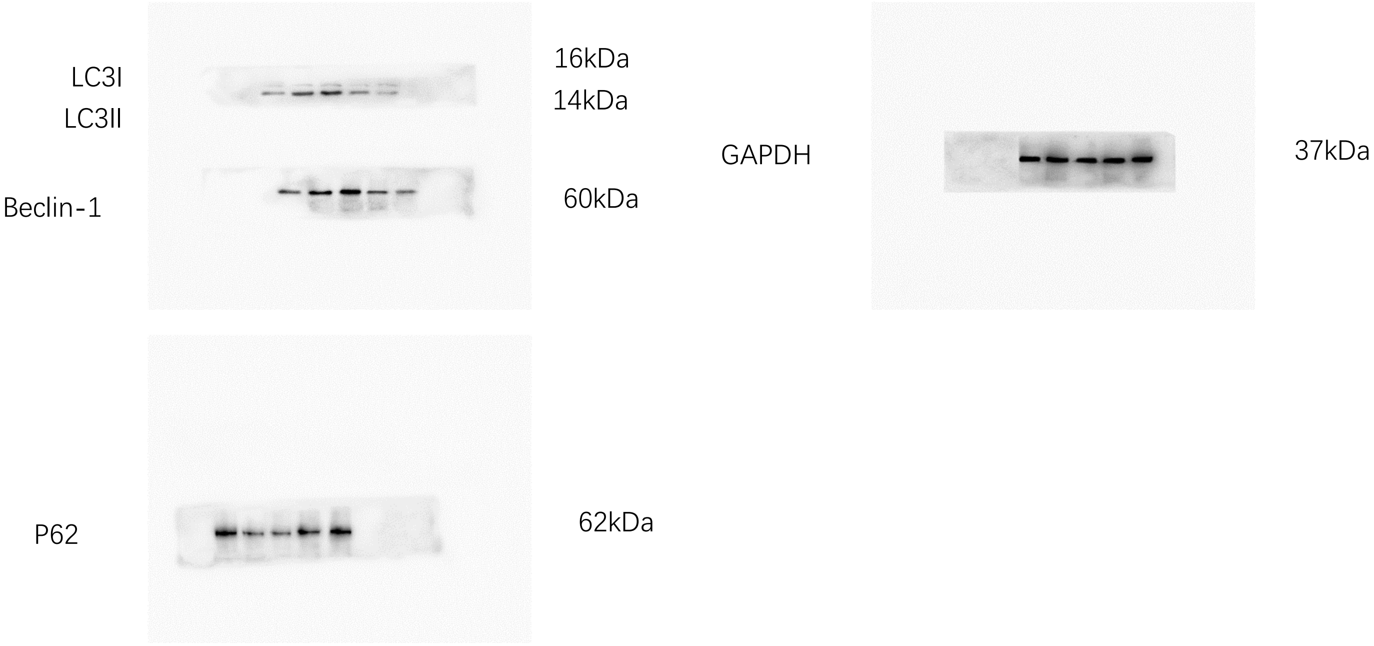

Supplementary Figure 7 – Related to Supplementary Fig. 2a.

a A western blot analysis was carried out on IL-1β-induced C28/I2 cells at different time points (0, 6, 12, 24, and 48 h) with antibodies directed at LC3, Beclin1 and p62. The autophagic activity of chondrocytes was increased in the early stage after IL-1β treatment, significantly inhibited after 24 h, and then remained inhibited.
