# Supplementary material for: Four-octyl itaconate improves osteoarthritis by enhancing autophagy in chondrocytes via PI3K/AKT/mTOR signalling pathway inhibition
Source: Commun Biol. 2022 Jun 29;5:641. doi: 10.1038/s42003-022-03592-6 (PMC9242998; doi:10.1038/s42003-022-03592-6)
Supplement: Supplementary file 3 — Description of Additional Supplementary Files [file 42003_2022_3592_MOESM3_ESM.pdf]

Description of Additional Supplementary

Files File name: Supplementary Data 1

Description: All source data underlying the graphs presented in the main figures.
