# Supplementary material for: Four-octyl itaconate improves osteoarthritis by enhancing autophagy in chondrocytes via PI3K/AKT/mTOR signalling pathway inhibition
Source: Commun Biol. 2022 Jun 29;5:641. doi: 10.1038/s42003-022-03592-6 (PMC9242998; doi:10.1038/s42003-022-03592-6)
Supplement: Supplementary file 5 — Reporting Summary [file 42003_2022_3592_MOESM5_ESM.pdf]

nature portfolio

Corresponding author(s): Xiaozhong Zhou

Last updated by author(s): Apr 24, 2022

Reporting Summary

Nature Portfolio wishes to improve the reproducibility of the work that we publish. This form provides structure for consistency and transparency in reporting. For further information on Nature Portfolio policies, see our Editorial Policies and the Editorial Policy Checklist.

Statistics

For all statistical analyses, confirm that the following items are present in the figure legend, table legend, main text, or Methods section.

n/a	Confirmed	
☐	☒	The exact sample size (*n*) for each experimental group/condition, given as a discrete number and unit of measurement
☐	☒	A statement on whether measurements were taken from distinct samples or whether the same sample was measured repeatedly
☐	☒	The statistical test(s) used AND whether they are one- or two-sided<br>*Only common tests should be described solely by name; describe more complex techniques in the Methods section.*
☐	☒	A description of all covariates tested
☐	☒	A description of any assumptions or corrections, such as tests of normality and adjustment for multiple comparisons
☐	☒	A full description of the statistical parameters including central tendency (e.g. means) or other basic estimates (e.g. regression coefficient) AND variation (e.g. standard deviation) or associated estimates of uncertainty (e.g. confidence intervals)
☐	☒	For null hypothesis testing, the test statistic (e.g. F, t, r) with confidence intervals, effect sizes, degrees of freedom and P value noted<br>*Give P values as exact values whenever suitable.*
☐	☒	For Bayesian analysis, information on the choice of priors and Markov chain Monte Carlo settings
☐	☒	For hierarchical and complex designs, identification of the appropriate level for tests and full reporting of outcomes
☐	☒	Estimates of effect sizes (e.g. Cohen's *d*, Pearson's *r*), indicating how they were calculated

Our web collection on statistics for biologists contains articles on many of the points above.

Software and code

Policy information about availability of computer code

Data collection	N/A
Data analysis	SPSS17.0 statistical software

For manuscripts utilizing custom algorithms or software that are central to the research but not yet described in published literature, software must be made available to editors and reviewers. We strongly encourage code deposition in a community repository (e.g. GitHub). See the Nature Portfolio guidelines for submitting code & software for further information.

Data

Policy information about availability of data

All manuscripts must include a data availability statement. This statement should provide the following information, where applicable:

- Accession codes, unique identifiers, or web links for publicly available datasets
- A description of any restrictions on data availability
- For clinical datasets or third party data, please ensure that the statement adheres to our policy

The datasets generated and/or analyzed during the current study are available from the corresponding author under an reasonable request. There is no restriction on data availability.

Field-specific reporting

Please select the one below that is the best fit for your research. If you are not sure, read the appropriate sections before making your selection.

☒ Life sciences ☐ Behavioural & social sciences ☐ Ecological, evolutionary & environmental sciences

For a reference copy of the document with all sections, see nature.com/documents/nr-reporting-summary-flat.pdf

Life sciences study design

All studies must disclose on these points even when the disclosure is negative.

Sample size	Sample size were provided in the manuscript. The sample size were determined based on literature and standard experimental design. We did not perform sample size calculation
Data exclusions	No data was excluded
Replication	The experimental results were repeated three times.
Randomization	Give animals the numbers, then use software random groups
Blinding	The investigators were blinded to group allocation during data collection and analysis.

Reporting for specific materials, systems and methods

We require information from authors about some types of materials, experimental systems and methods used in many studies. Here, indicate whether each material, system or method listed is relevant to your study. If you are not sure if a list item applies to your research, read the appropriate section before selecting a response.

Materials & experimental systems

n/a	Involved in the study	
☐	☒	Antibodies
☐	☒	Eukaryotic cell lines
☒	☐	Palaeontology and archaeology
☐	☒	Animals and other organisms
☒	☐	Human research participants
☒	☐	Clinical data
☒	☐	Dual use research of concern

Methods

n/a	Involved in the study	
☒	☐	ChIP-seq
☐	☒	Flow cytometry
☒	☐	MRI-based neuroimaging

Antibodies

Antibodies used	anti-PI3K (1:1000, Abcam, ab32089), anti-Bcl-2 (1:1000, Abcam, ab32124), anti-AKT (1:2000, Abcam, ab8805), anti-pAKT (1:500, Abcam, ab38449), anti-BAX (1:1000, Abcam, ab53154), anti-LC3B(1:500, Abcam, ab221794), anti-p62 (1:2000, Abcam, ab91526), anti-mTOR (1:1000, Abcam, ab32028), anti-cleaved caspase 3 (1:1000, Abcam, ab2302), and anti-pro-caspase 3 (1:1000, Abcam, ab205733).
Validation	Antibodies were validated in mouse, rat and cell lines.

Eukaryotic cell lines

Policy information about cell lines

Cell line source(s)	C28/I2
Authentication	C28/I2 chondrocytes were acquired from HyClone (USA)
Mycoplasma contamination	N/A
Commonly misidentified lines (See ICLAC register)	N/A

Animals and other organisms

Policy information about studies involving animals; ARRIVE guidelines recommended for reporting animal research

Laboratory animals	5-week-old male Sprague Dawley rats (250-300g)
Wild animals	N/A
Field-collected samples	N/A
Ethics oversight	All animal experiments were conformed to the U.S. National Institutes of Health Guidelines (NIH Publication No. 85-23, revised 1996), and the protocol was approved by the Ethics Committee of the Second Hospital of Jiaxing (JXEY-2020JX051).

Note that full information on the approval of the study protocol must also be provided in the manuscript.

Flow Cytometry

Plots

Confirm that:

☒ The axis labels state the marker and fluorochrome used (e.g. CD4-FITC).

☒ The axis scales are clearly visible. Include numbers along axes only for bottom left plot of group (a 'group' is an analysis of identical markers).

☒ All plots are contour plots with outliers or pseudocolor plots.

☒ A numerical value for number of cells or percentage (with statistics) is provided.

Methodology

Sample preparation	C28/I2 chondrocytes were acquired from HyClone (USA).Approximately 10 μL fluorescently-labeled Annexin V reagent and 5 μL PI reagent were added into the 1.5 mL centrifuge tubes, following collecting the chondrocytes by centrifugation at 300 g for 10 min. After incubating for 10 min at room temperature in the dark, approximately 200 μL of solution containing cells was mixed with 2 mL PBS in the flow tubes, which was tested by the flow cytometry (Thermo Fisher Scientific, Waltham, USA).
Instrument	Flow cytometry (Thermo Fisher Scientific, Waltham, USA)
Software	Cellquest Pro
Cell population abundance	C28/I2 chondrocytes were acquired from HyClone (USA)
Gating strategy	Fluorescence compensation and negative controls to determine the gate.

☒ Tick this box to confirm that a figure exemplifying the gating strategy is provided in the Supplementary Information.
